# Planting the seeds for a forest of RNAi pathways

**DOI:** 10.1371/journal.pbio.3002279

**Published:** 2023-08-16

**Authors:** Peter Sarkies

**Affiliations:** Department of Biochemistry, University of Oxford, Oxford, United Kingdom

## Abstract

This Perspective looks back at a classic PLOS Biology paper that provided an early glimpse of the complexity of RNAi pathways in plants and how it acted as a springboard for further exploration of this extravagant universe.

## Teaser

Cells from most eukaryotic species make several different types of small interfering RNAs. Pioneering work in plants, published in *PLOS Biology* almost 20 years ago, established a framework to understand how multiple RNA interference pathways can regulate the genome in parallel.

This article is part of the *PLOS Biology* 20th Anniversary Collection.

It’s a cliché repeated at the beginning of many papers that plants “can’t run.” Plant researchers, on the other hand, move very fast indeed, often leaving animal biologists far behind as they push molecular biology into exciting new territory. Remarkably, despite the extraordinary evolutionary distance and the entirely separate evolution of multicellularity in the two groups, many of the discoveries made in plants later turn out to be conceptually similar or even homologous in animals.

RNA interference (or RNAi) is a striking example where plants “got there first,” uncovering a transformative molecular mechanism that turned out to be essential for animals too. The basic process of RNAi involves a small noncoding RNA, which, in conjunction with a member of a family of proteins known as Argonautes, regulates a target mRNA, leading to its silencing [1]. The source of the small RNA is often, but not always, the activity of an endonuclease known as Dicer [2]. Dicer cuts double-strand RNA to release short duplex RNAs usually around 22 nucleotides in length, which are then fed into Argonaute proteins for silencing [3].

The central process of RNAi was first discovered in plants and characterised as a form of antiviral defence. The double-strand RNA could come directly from RNA viruses; single-strand RNA viruses will produce double-strand RNAs as part of their replication cycle [1]. Later, RNAi was shown to act in regulation of endogenous genes and to have many marvellous properties. One such property is the ability for cells to amplify RNAi responses using a genomically encoded RNA-dependent RNA polymerase, similar to the ones that RNA viruses use to replicate [4]. RNAi can also spread between tissues to communicate the threat of infection and silencing signals for endogenous gene regulation [5]. Most strikingly, RNAi can initiate silencing that can be inherited transgenerationally: transgenerational epigenetic inheritance where a gene expression change can be inherited for many generations completely without change in the sequence of DNA bases [6]. All these features were discovered in plants and later shown to be conserved in animals.

**Fig 1:**
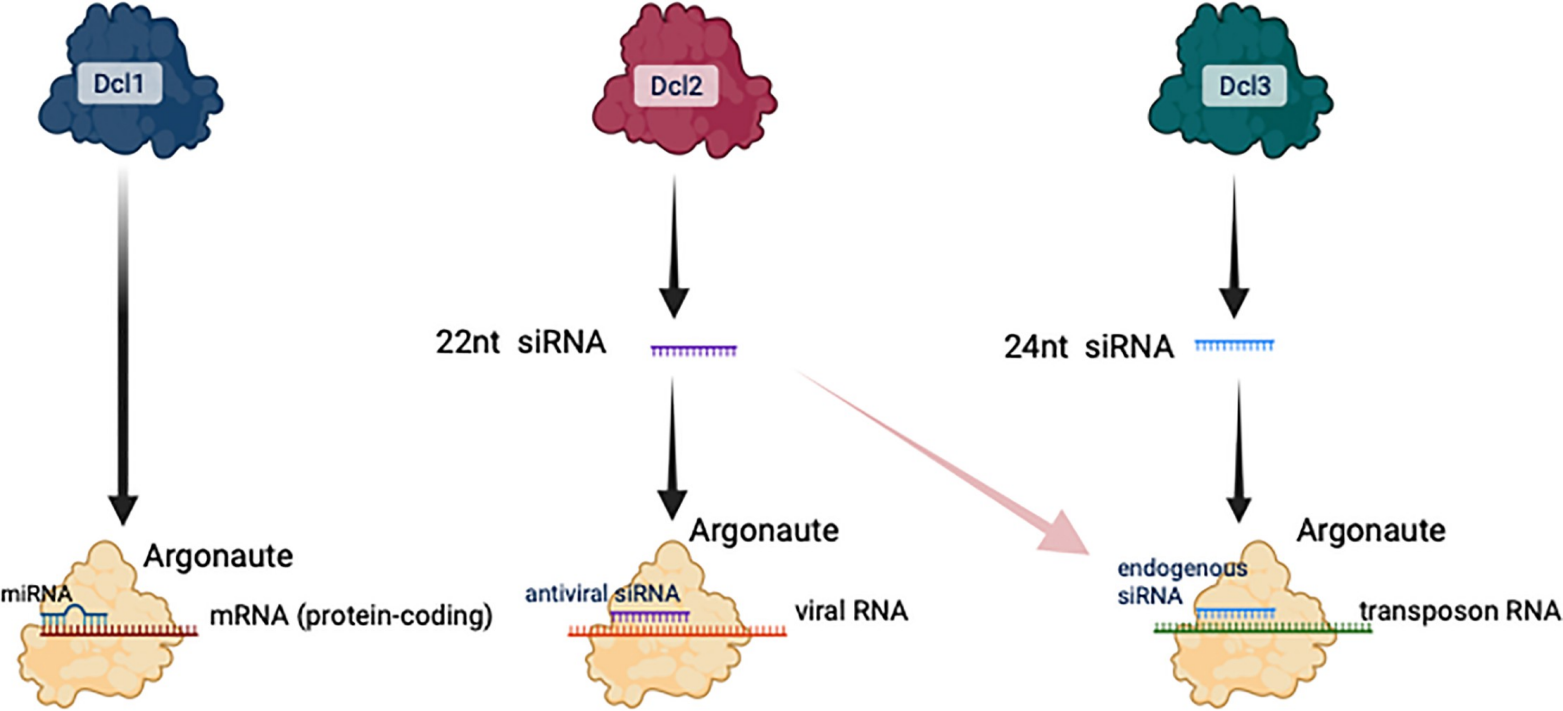
A simplified schematic of the model proposed by Xie and colleagues [7] for how different Dicer paralogues contribute to RNAi in plants. Multiple Argonaute proteins are required for the silencing. Later discoveries included the participation of 22-nucleotide siRNAs in endogenous RNAi pathways, indicated by the pink arrow. Nevertheless, the overall framework presented here has largely been supported by subsequent studies. Figure prepared with Biorender.com.

One of the key properties that underpins all this flamboyance is the existence of several parallel mechanisms to process and partition the many pathways of RNAi. The most conceptually straightforward way in which this can occur is through paralogues with dedicated specificity. Early on, it was recognised that plants contain several Dicer and RNA-dependent RNA polymerase paralogues; however, it took a major advance, published in *PLOS Biology* in 2004, to clearly demonstrate that these paralogues really were at the heart of diversity in RNAi [7]. Xie and colleagues analysed mutants in three of the four Dicer paralogues (DCL1, 2, and 3) and three RNA-dependent RNA polymerases (RDR1, RDR2, and RDR6). This enabled them to delineate the genetic requirements of three separate pathways involving small noncoding RNAs. First, they showed that microRNAs, which are a class of genomically encoded small noncoding RNAs that bind to Argonautes and are involved in regulating canonical protein-coding genes, are dependent on DCL1 but do not require any RNA-dependent RNA polymerases. Second, they discovered that the antiviral pathway of RNAi relies on DCL2 and RDR2, but not RDR1. Finally, they showed that endogenous RNAi involved DCL3 [7]. A simplified diagram of this model is presented in Fig 1.

In addition to showing distinct genetic requirements for these pathways, the paper also made some important observations that turned out to be prescient in understanding RNAi pathway diversity across eukaryotes. In 2004, the high-throughput sequencing methods that are so ubiquitous now were not readily available. Nevertheless, using standard cloning and sequencing techniques, Xie and colleagues assayed over 1,000 different endogenous small noncoding RNAs corresponding to the DCL3 pathway. They showed that these RNAs had a modal length of 24 nucleotides, contrasting to the modal length of 22 nucleotides that characterises antiviral small noncoding RNAs [1]. Different types of small noncoding RNAs thus were shown to have different biogenesis and different sequence properties, something that has turned out to be an extremely general feature of RNAi and related small RNA pathways. Additionally, it was possible to discern from this analysis that DCL3-derived endogenous small noncoding RNAs were largely similar in sequence to transposable elements. Indeed, they also determined that cytosine DNA methylation at CHH and CHG sites, key in silencing transposable elements, was lost in mutants lacking these endogenous small noncoding RNAs, hinting at a potential transcriptional mechanism whereby small noncoding RNAs could contribute to transposable element silencing, showing that small noncoding RNAs might have nuclear as well as cytoplasmic functionality [7].

Many gaps remained to be filled in. We now know that RDR1, RDR2, and RDR6 all contribute to viral defence to some extent [8]. A fourth Dicer, DCL4, was not examined by Xie and colleagues, and this turns out to produce 21-nucleotide siRNAs [9]. Moreover, there are some endogenous small noncoding RNAs that are 22 nucleotides long and are produced by DCL2, similar to the antiviral pathway but targeting endogenous genes. The clear delineation of dicers into different pathways is not always seen in other organisms. For example, in the nematode *C*. *elegans*, the one dicer enzyme carries out antiviral RNAi, endogenous RNAi, and microRNA biogenesis, with cofactors and different downstream Argonaute proteins delimiting the pathways [10]. Indeed, the fact that some RNAi pathways share certain components has been shown to be an important way to regulate their activity [11]. Nevertheless, categorising the size of small RNAs, their genetic components, and whether they are microRNAs, endogenous siRNAs, and exogenous siRNAs has proved to be a highly productive general framework for understanding these fascinating molecular pathways.

From a personal perspective, my interest in the evolutionary aspects of small noncoding RNA pathways can trace its descent to the pioneering experiments of Xie and colleagues. While they were studying RNAi in one species, the existence of multiple pathways coexisting often in the same cell emphasises the evolutionary plasticity of RNAi. The specialisation of the paralogues implies that a common ancestral RNAi pathway, perhaps involving one Dicer, one RNA-dependent RNA polymerase, and one Argonaute, can evolve rapidly, with new pathways such as the transposon-controlling piRNAs emerging in animals [12], and new functions such as in protein-coding gene regulation evolving independently in distinct lineages. This plasticity can also encompass gene loss, as seen in the frequent loss of RNA-dependent RNA polymerases across animals (despite it being ancestral) [13], and the ability of RNAi pathways to compensate for frequent loss of piRNAs in nematodes [14]. Of course, what we still don’t know is why some small noncoding RNA pathways, particularly piRNAs and RNAi pathways that use RNA-dependent RNA polymerases, appear to evolve so rapidly compared to other highly conserved gene regulatory pathways. Perhaps this will take another pioneering *PLOS Biology* paper to unravel!

## References

[1] Baulcombe D. RNA silencing in plants. Nature. 2004;431:356–363. doi: 10.1038/nature02874 15372043

[2] Vergani-Junior CA, Tonon-Da-Silva G, Dinçer Inan M, Mori MA. DICER: structure, function, and regulation. Biophys Rev. 1:3. doi: 10.1007/s12551-021-00902-w 35059029 PMC8724510

[3] Swarts DC, Makarova K, Wang Y, Nakanishi K, Ketting RF, Koonin EV, et al. The evolutionary journey of Argonaute proteins. Nat Struct Mol Biol. 2014;21:743–753. doi: 10.1038/nsmb.2879 25192263 PMC4691850

[4] Fukudome A, Singh J, Mishra V, Reddem E, Martinez-Marquez F, Wenzel S, et al. Structure and RNA template requirements of Arabidopsis RNA-DEPENDENT RNA POLYMERASE 2. Proc Natl Acad Sci U S A. 2021;118:e2115899118. doi: 10.1073/pnas.2115899118 34903670 PMC8713982

[5] Sarkies P, Miska EA. Small RNAs break out: The molecular cell biology of mobile small RNAs. Nat Rev Mol Cell Biol. 2014:525–535. doi: 10.1038/nrm3840 25053358

[6] Cecere G. Small RNAs in epigenetic inheritance: from mechanisms to trait transmission. FEBS Lett. 2021:2953–2977. doi: 10.1002/1873-3468.14210 34671979 PMC9298081

[7] Xie Z, Johansen LK, Gustafson AM, Kasschau KD, Lellis AD, Zilberman D, et al. Genetic and Functional Diversification of Small RNA Pathways in Plants. PLoS Biol. 2004;2:e104. doi: 10.1371/journal.pbio.0020104 15024409 PMC350667

[8] Hua X, Berkowitz ND, Willmann MR, Yu X, Lyons E, Gregory BD. Global analysis of rna-dependent rna polymerase-dependent small rnas reveals new substrates and functions for these proteins and sgs3 in arabidopsis. Noncoding RNA. 2021;7:1–19. doi: 10.3390/ncrna7020028 33925339 PMC8167712

[9] Parent JS, Bouteiller N, Elmayan T, Vaucheret H. Respective contributions of Arabidopsis DCL2 and DCL4 to RNA silencing. Plant J. 2015;81:223–232. doi: 10.1111/tpj.12720 25376953

[10] Duchaine TF, Wohlschlegel JA, Kennedy S, Bei Y, Conte D, Pang KM, et al. Functional proteomics reveals the biochemical niche of C. elegans DCR-1 in multiple small-RNA-mediated pathways. Cell. 2006;124:343–354. doi: 10.1016/j.cell.2005.11.036 16439208

[11] Houri-Ze’evi L, Korem Y, Sheftel H, Faigenbloom L, Toker IA, Dagan Y, et al. A Tunable Mechanism Determines the Duration of the Transgenerational Small RNA Inheritance in C. elegans. Cell. 2016;165:88–99. doi: 10.1016/j.cell.2016.02.057 27015309

[12] Brennecke J, Aravin AA, Stark A, Dus M, Kellis M, Sachidanandam R, et al. Discrete Small RNA-Generating Loci as Master Regulators of Transposon Activity in Drosophila. Cell. 2007;128:1089–1103. doi: 10.1016/j.cell.2007.01.043 17346786

[13] Lewis SH, Quarles KA, Yang Y, Tanguy M, Frézal L, Smith SA, et al. Pan-arthropod analysis reveals somatic piRNAs as an ancestral defence against transposable elements. Nat Ecol Evol. 2018;2:174–181. doi: 10.1038/s41559-017-0403-4 29203920 PMC5732027

[14] Sarkies P, Selkirk ME, Jones JT, Blok V, Boothby T, Goldstein B, et al. Ancient and Novel Small RNA Pathways Compensate for the Loss of piRNAs in Multiple Independent Nematode Lineages. PLoS Biol. 2015;13(2):e1002061. doi: 10.1371/journal.pbio.1002061 25668728 PMC4323106

